# A Novel Immunochromatographic Test Applied to a Serological Survey of Japanese Encephalitis Virus on Pig Farms in Korea

**DOI:** 10.1371/journal.pone.0127313

**Published:** 2015-05-20

**Authors:** Go-Woon Cha, Eun Ju Lee, Eun-Joo Lim, Kang Suk Sin, Woo Won Park, Doo Young Jeon, Myung Guk Han, Won-Ja Lee, Woo-Young Choi, Young Eui Jeong

**Affiliations:** 1 Division of Arboviruses, National Institute of Health, Korea Centers for Disease Control and Prevention, Cheongju-si, Chungcheongbuk-do, Korea; 2 Gangwon Institute of Health and Environment, Shibuk-ro, Chuncheon-si, Gangwon-do, Korea; 3 Chungbuk Institute of Health and Environment, Cheongju-si, Chungcheongbuk-do, Korea; 4 Gyeongbuk Institute of Health and Environment, Yeongcheon-si, Gyeongsangbuk-do, Korea; 5 Jeonnam Institute of Health and Environment, Muan-gun, Jeollanam-do, Korea; 6 Department of Biomedical Sciences, Graduate School of Hallym University, Chuncheon-si, Gangwon-do, Korea; University of California Davis, UNITED STATES

## Abstract

Among vertebrate species, pigs are a major amplifying host of Japanese encephalitis virus (JEV) and measuring their seroconversion is a reliable indicator of virus activity. Traditionally, the hemagglutination inhibition test has been used for serological testing in pigs; however, it has several limitations and, thus, a more efficient and reliable replacement test is required. In this study, we developed a new immunochromatographic test for detecting antibodies to JEV in pig serum within 15 min. Specifically, the domain III region of the JEV envelope protein was successfully expressed in soluble form and used for developing the immunochromatographic test. The test was then applied to the surveillance of Japanese encephalitis (JE) in Korea. We found that our immunochromatographic test had good sensitivity (84.8%) and specificity (97.7%) when compared with an immunofluorescence assay used as a reference test. During the surveillance of JE in Korea in 2012, the new immunochromatographic test was used to test the sera of 1,926 slaughtered pigs from eight provinces, and 228 pigs (11.8%) were found to be JEV-positive. Based on these results, we also produced an activity map of JEV, which marked the locations of pig farms in Korea that tested positive for the virus. Thus, the immunochromatographic test reported here provides a convenient and effective tool for real-time monitoring of JEV activity in pigs.

## Introduction

Japanese encephalitis virus (JEV), belonging to the genus *Flavivirus* in the family *Flaviviridae*, is a causative pathogen of acute encephalitis in humans [1]. The JEV genome comprises a single-stranded, positive-sense RNA molecule and encodes a single polyprotein, which is processed into three structural proteins and seven non-structural proteins with un-translated regions at the 5′ and 3′ ends [1]. Of these, the envelope protein (E) is involved in viral attachment, neutralizing antibody response, and protective immunity; thus, it is the major target antigen for vaccine development and laboratory diagnosis.

In nature, JEV is transmitted via a cycle that includes (a) infection of pigs and water birds by horizontal transmission from mosquito vectors, (b) infection of certain mosquitoes by horizontal transmission from vertebrate amplifiers, and (c) vertical transmission from adult female mosquitoes to their progeny [2]. Humans and horses are infected incidentally in this cycle and considered dead-end hosts because the level of viremia is insufficient to infect the mosquito vector. According to the latest report in 2011 [3], more than 67,000 cases of Japanese encephalitis (JE), with 20–30% fatality, occur annually throughout Asia and the Pacific regions.

Vaccination of humans and the amplifying host, pigs, is the most effective method for preventing JE outbreaks; thus, several vaccines have been developed for humans and pigs [4]. Additionally, monitoring JEV activity in mosquito vectors and domestic pigs enables health authorities to accurately predict risk and conduct timely vector control measures where risks are detected. In practice, some countries monitor seroconversion in sentinel animals such as pigs, goats, and chickens [5–12]. In Korea, the National Institute of Health (KNIH) monitors JEV transmission by determining the levels of antibody in slaughtered pigs during July and October every year as part of the national JE Epidemic Forecast Program initiated in 1975 [13].

Traditionally, the hemagglutination inhibition (HI) test has been used for antibody detection in pig serum [5–11] and, more recently, indirect enzyme-linked immunosorbent assays (ELISAs) have been developed and partially used during surveillance [12, 14, 15]. For the HI test, fresh erythrocytes are ideally harvested from female geese at 2–3 week intervals and hundreds of suckling mice are injected with the virus and killed to obtain the antigen [16], which is a somewhat controversial practice in relation to laboratory animal welfare. In addition, the test involves more than 12 reagents that must be made in-house, some of which are pH-sensitive and highly toxic. Furthermore, it is often difficult to compare test results between laboratories because of variations in goose erythrocytes, mouse brain-derived antigens, and in-house reagents. While two specific ELISAs have been developed and evaluated [14, 15], recombinant antigens should replace mouse brain-derived or chicken embryo-derived antigens in these tests to minimize test variation. Immunofluorescent assays (IFAs) are an alternative tool for detecting antibodies to JEV in pig serum, and they have been effective for the clinical diagnosis of flaviviruses such as West Nile virus, yellow fever virus, and JEV [17–19]. The immunochromatographic assay has been used for diagnosis of contagious human diseases since the 1980s and has recently been extended to other fields because it is easy to use, has a short running time (within 20 min), and produces results that can be read by eye. For example, the technique is now used to detect antigens or antibodies of animal viruses such as avian influenza virus [20], porcine reproductive and respiratory syndrome virus [21], and porcine circovirus-2 [22].

In order to improve the surveillance of JE, we developed an immunochromatographic test for detecting antibodies to JEV in pigs and successfully applied it in national JE surveillance conducted in South Korea.

## Materials and Methods

### Ethics statement

The serological survey of JE antibodies in domestic pigs has been conducted with the permission from the Ministry of Health & Welfare in Korea and according to the Act on the Prevention and Control of Infectious Diseases. In this study, ethical approval for animal experimentation was not required because we used serum samples from pigs that were slaughtered for meat production. The names of the slaughterhouses from which samples were collected are as follows: Bukyong pork producers associations’ abattoir (35.23736°N, 128.909012°E), Namwon Jeil Food abattoir (35.40998°N, 127.311738°E), Naju Chukhyup abattoir (34.981645°N, 126.699679°E), Minsok LPC abattoir (36.266698°N, 128.572826°E), Samse abattoir (35.929371°N, 128.929614°E), Goryeong Nonghyup abattoir (35.798793°N, 128.392947°E), Gongju Food abattoir (36.491352°N, 127.136021°E), and Cheorwon Hanyang abattoir (38.187466°N, 127.316437°E). The respective owners of the private slaughterhouses granted permission for serum to be collected on their property.

### Virus and cell line

The Nakayama strain of JEV was purchased from the National Collection of Pathogen Viruses (London, UK). It was used as a template for the cloning and expression of the recombinant protein in *Escherichia coli*. BHK-21 cells (ATCC, CCL-10) were used for virus propagation and for producing antigen slides.

### Construction of expression vectors for the E gene of JEV

**Fig 1:**
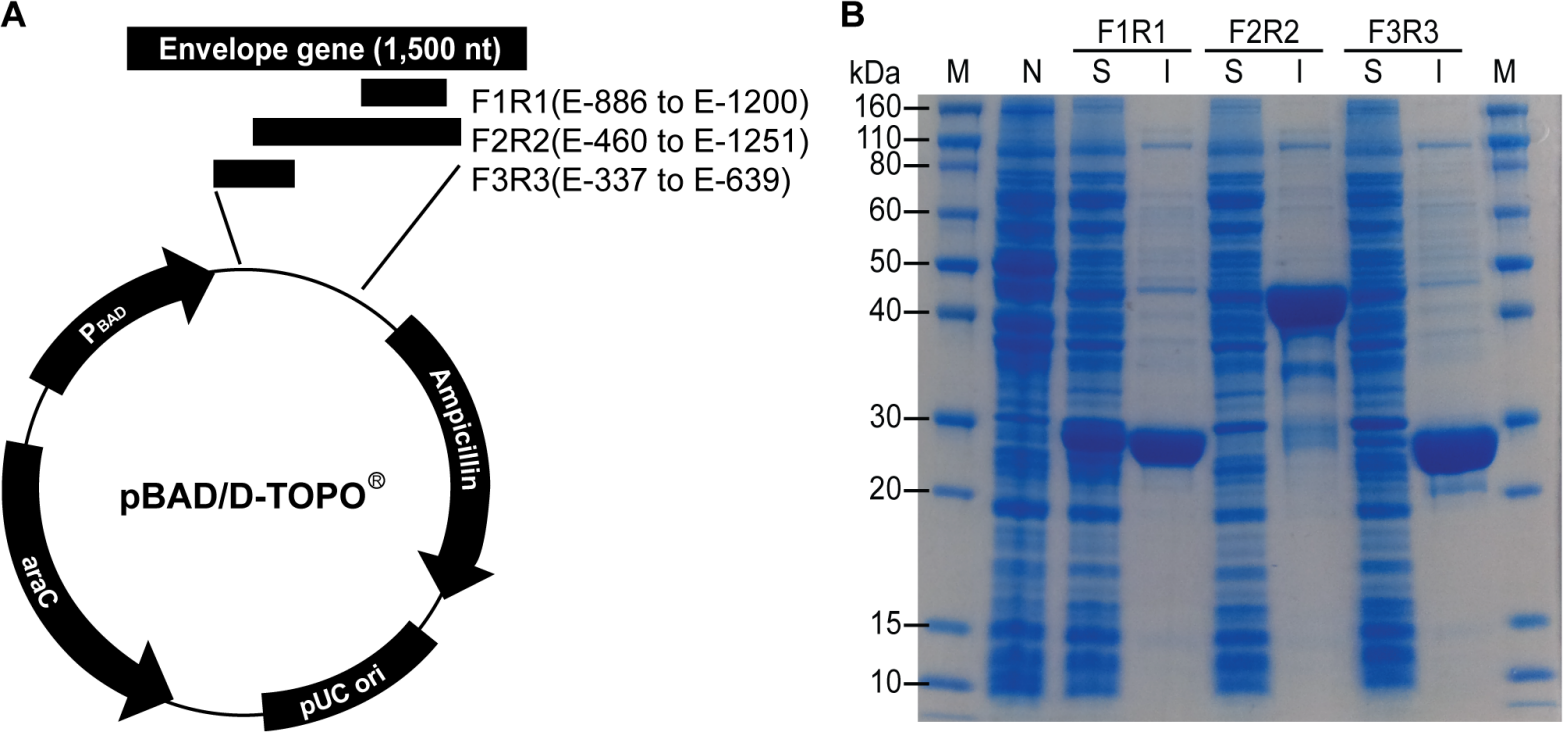
Expression of the envelope (E) protein of Japanese encephalitis virus (JEV). (A) Cloning strategy for the expression of the E protein of JEV and (B) gel electrophoresis of the expressed protein. Three different fragments of the E gene from JEV were cloned into a pBAD/D-TOPO vector and over-expressed in *Escherichia coli* cells. The supernatant and pellet of lysed cells were analyzed by gel electrophoresis. Lanes: M, molecular weight markers; N, negative control (non-induced cell); S, soluble fraction (supernatant); I, insoluble fraction (pellet).

RNA was extracted from the stock virus (Nakayama strain) using the QIAamp viral RNA mini kit (QIAGEN, Hilden, Germany). The purified RNA was used as template for cDNA synthesis using the SuperScript III first-strand synthesis system (Life Technologies, Carlsbad, CA, USA) with primer JE-1494R (Table 1). Three fragments of the E gene were amplified from the synthesized cDNA with each primer pair (F1R1, F2R2, and F3R3) using AccuPrime *Pfx* DNA polymerase (Life Technologies) (Table 1). Among the fragments, the F1R1 corresponded to the amino acid residues 296–400 (domain III) in the E protein. Other fragments were selected to express more antigenic moieties or hydrophilic regions. The amplified products were visualized using agarose gel electrophoresis, purified using a QIAquick Gel Extraction kit (QIAGEN), and ligated into the pBAD102/D-TOPO vector (Life Technologies) (Fig 1). The constructed plasmids were incorporated into One Shot TOP10 competent cells and the resultant transformants were selected on LB plates supplemented with 50 μg/mL of ampicillin. Finally, the positive colonies harboring the correct insert were confirmed by PCR and sequencing analysis. These colonies were allowed to grow further in LB medium, mixed with sterile glycerol, and stored at −70°C until further use.

**Table 1:** Primers used for cDNA synthesis and amplification of the envelope (E) gene of Japanese encephalitis virus.

Name	Sequence (5' to 3')	Position^a^	Use
JE_1494R	CACATTGGTCGCTAAGAACACGAGC	1494–1470	cDNA synthesis
F1	CACCCTG AAA GGC ACA ACC TA	886–902	Amplification
R1	TCC AGC CTT GTG CCA ATG GTG	1200–1180	
F2	CACC AAT TAT TCA GCG CAA GTT GG	460–479	Amplification
R2	CAG TCT TTG AGC TCC CTT CAA AGT C	1251–1227	
F3	CACC ATT GAC ACA TGT GCA AAA TTC	337–357	Amplification
R3	GAC CAA AAA TGA CTT TGA CC	639–620	

^a^The nucleotide position was based on the E gene of the Nakayama strain (GenBank U70413). The sequence of CACC at the 5' end of the primer was added for directional cloning according to the manufacturer's instructions.

### Expression and purification of recombinant proteins

Each transformant was grown in 50 mL of LB medium containing ampicillin (50 μg/mL) overnight at 37°C with vigorous shaking (250 rpm). The following morning, 1 mL of the overnight culture was added to 100 mL of LB medium (ampicillin^+^) and further cultured at 37°C with vigorous shaking (250 rpm). When the cell density reached mid-log phase (OD_600_ = ~0.5), 100 μL of arabinose stock solution (20% in distilled water) was added to each culture to a final concentration of 0.02%. The cells were further cultured for 3 h at 37°C with vigorous shaking in order to induce protein expression. Subsequently, the culture was divided into two aliquots of 50 mL and harvested by centrifugation (3,500 rpm for 25 min at 8°C). To disrupt cells, 3 mL of BugBurster Protein Extraction reagent (Novagen, Germany) was added with gentle shaking at room temperature for 20 min. We used gel electrophoresis and western blotting to analyze the supernatant. The remaining pellet was treated with 800 μL of LDS sample buffer (141 mM Tris base, 2% lithium dodecyl sulfate, 10% glycerol, 0.51 mM EDTA, 0.22 mM SERVA Blue G, 0.175 mM Phenol Red, pH 8.5) and incubated at 70°C for 10 min to accelerate cell bursting. Following centrifugation, the supernatant was collected and analyzed by gel electrophoresis and western blotting.

The soluble protein fused with the C-terminal polyhistidine (6×His) tag was purified in native conditions using the ProBond Purification system (Life Technologies) according to the manufacturer’s protocol. Subsequently, the protein was eluted with native elution buffer containing 250 mM imidazole. The purity of the eluted fractions was analyzed by gel electrophoresis. The appropriate fractions were pooled and dialyzed against phosphate-buffered saline. The concentration of the protein was determined based on the Bradford method using the Bio-Rad Protein assay (Bio-Rad, Hercules, CA, USA). For large scale production of the protein, 200 mL of culture was harvested, lysed as described, and purified using a HisTrap HP column in the ÄKTA prime plus instrument (GE Healthcare Life Sciences, Pittsburgh, PA, USA).

### Gel electrophoresis and western blotting of the recombinant proteins

Protein samples were analyzed using the NuPAGE Bis-Tris Gel Electrophoresis system (Life Technologies). For western blotting, proteins that had been separated by gel electrophoresis were transferred onto a PVDF membrane using an iBlot 2 Gel Transfer Device (Life Technologies). The blots were blocked for 1 h with TBS-Tween (TBST; USB Corporation, Cleveland, OH, USA) containing 5% skim milk and then reacted for 1 h with a 1:100 dilution of JEV-positive human sera in the same buffer. The JEV-positive sera were pooled from five patients, which were diagnosed by using a commercial ELISA (InBios, Seattle, WA, USA) and an in-house IgG IFA [23] at the Division of Arboviruses in the KNIH. The blot was washed three times in TBST (10 min per wash) and incubated for 1 h in a 1:2,500 dilution of horseradish peroxidase-conjugated goat anti-human IgG (Jackson ImmunoResearch, Westgrove, PA, USA). After three further washes with TBST, the blot was developed using Pierce enhanced chemiluminescence detection reagents (Thermo Scientific, Waltham, MA, USA).

### Development of theimmunochromatographic test

**Fig 2:**
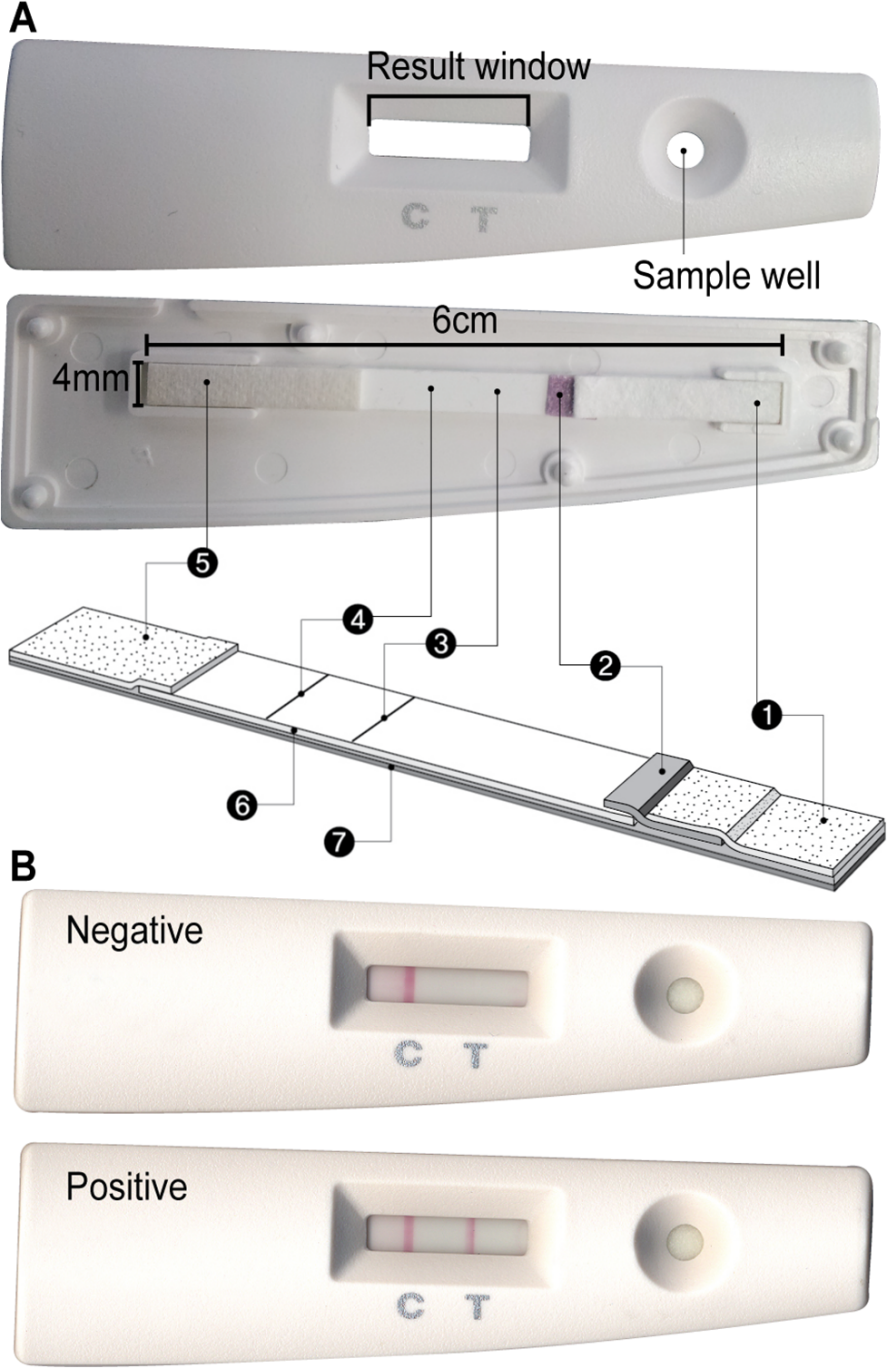
The immunochromatographic test. (A) Schematic diagram of the immunochromatographic test and (B) typical results obtained from testing pig serum. Labeled parts: 1, sample pad; 2, gold conjugation pad; 3, test line; 4, control line; 5, absorbance pad; 6, nitrocellulose membrane; 7, plastic backing.

The Division of Arboviruses at the KNIH developed the immunochromatographic test for detection of antibodies against JEV in pigs, and it was patented in Korea (Patent No. 10–0877913). In this study, the test kit was manufactured by a company (Wonkangbio, Gyeonggi, Korea) that received a non-exclusive license for the patent from the Korean Intellectual Property Office. The test kit used a colloidal gold-based lateral-flow immunoassay technique. A schematic diagram of the test is shown in Fig 2A. The highly purified F1R1 subunit of the JEV E protein described above was immobilized on the nitrocellulose membrane near the sample well (T line). Goat anti-mouse IgG antibodies were also immobilized on the nitrocellulose membrane near the absorbance pad (C line). The colloidal gold particles (40 nm diameter in solution) were conjugated with mouse anti-pig IgG antibodies (Jackson ImmunoResearch), soaked into a polyester pad, and dried at 37°C in an incubator. The membrane immobilized with the E protein and antibodies, the gold conjugation pad, the absorbance pad, and the pre-treated sample pad were glued onto plastic backing sheets (30 cm × 6 cm) and then cut into 4-mm-wide strips using an automatic cutter. Each strip was assembled on a plastic cassette and sealed with a desiccant (0.5 g) in an aluminum pouch. The test was stored at a broad temperature range (4–30°C) prior to use.

For testing, 10 μL of pig serum was added in a sample well and then 3 drops (90 μL) of running buffer were added to the sample well. The result was read by eye between 5–15 min after the addition of the running buffer. When the color red was shown on both T and C lines in the results window, the result was positive, and when red appeared only on the C line, the result was negative (Fig 2B).

### Sensitivity and specificity of the immunochromatographic test

For evaluating the test kit, 246 pig sera collected during surveillance in 2009–2010 were tested by IgG IFA as a reference test. To make antigen slides, BHK-21 cells were infected with JEV Nakayama strain, cultured for 15–17 h, and then the infected cells were dispensed to spotted glass slides [23]. Pig serum samples were diluted 1:16, added to slide wells for 30 min, washed with phosphate-buffered saline, and then reacted with fluorescein isothiocyanate-labeled goat anti-swine IgG antibodies (Jackson ImmunoResearch) for 30 min. Subsequently, the slides were examined under a fluorescence microscope (Axioskop 2 plus, Carl Zeiss, Germany) in a dark room. Sensitivity and specificity of the test kit were calculated using 112 JEV-positive and 134 JEV-negative samples.

### Application of the immunochromatographic test in the field

The prevalence of antibodies to JEV was monitored among slaughtered pigs in eight provinces of Korea from June to October 2012. At each slaughterhouse, a veterinarian randomly selected 10–20 pig sera from 1–2 piggeries. The vaccination history and farm address of pigs were recorded and only un-vaccinated pigs were included in the study. The specimens were immediately transported to a designated laboratory at the Institute of Health & Environment (IHE) in each of eight provinces and then subjected to the immunochromatographic test. To visualize the JEV-active locations in detail, we used the ArcGIS software (Redlands, CA, USA) to mark the latitude and longitude of the pig farms where pigs tested positive.

Prior to application in the field, a proficiency test was conducted in the eight provincial IHEs. The immunochromatographic test kits and five pig sera (three JEV-positive and two negative samples) were allocated to each laboratory. The test result was returned to the Division of Arboviruses at the KNIH within 10 days of receipt of the reagents.

## Results

### Cloning and expression of the recombinant E protein of JEV

Three expression vectors were successfully constructed with different positions and lengths in the E gene: pBAD-F1R1, pBAD-F2R2, and pBAD-F3R3 (Fig 1A). Similar to theoretical expectations computed using the Compute pI/Mw tool [24], the molecular weight of expressed proteins from pBAD-F1R1, pBAD-F2R2, and pBAD-F3R3 was 28.0, 45.2, and 27.7kDa, respectively (Fig 1B). However, all the proteins expressed from pBAD-F2R2 and pBAD-F3R3 were found only in insoluble fractions. Despite changing conditions such as arabinose concentration, culture time and temperature, and cell bursting methods, the proteins were expressed only in the cell pellet. Thus, only proteins expressed from pBAD-F1R1 were used for further analysis and development of the test kit.

**Fig 3:**
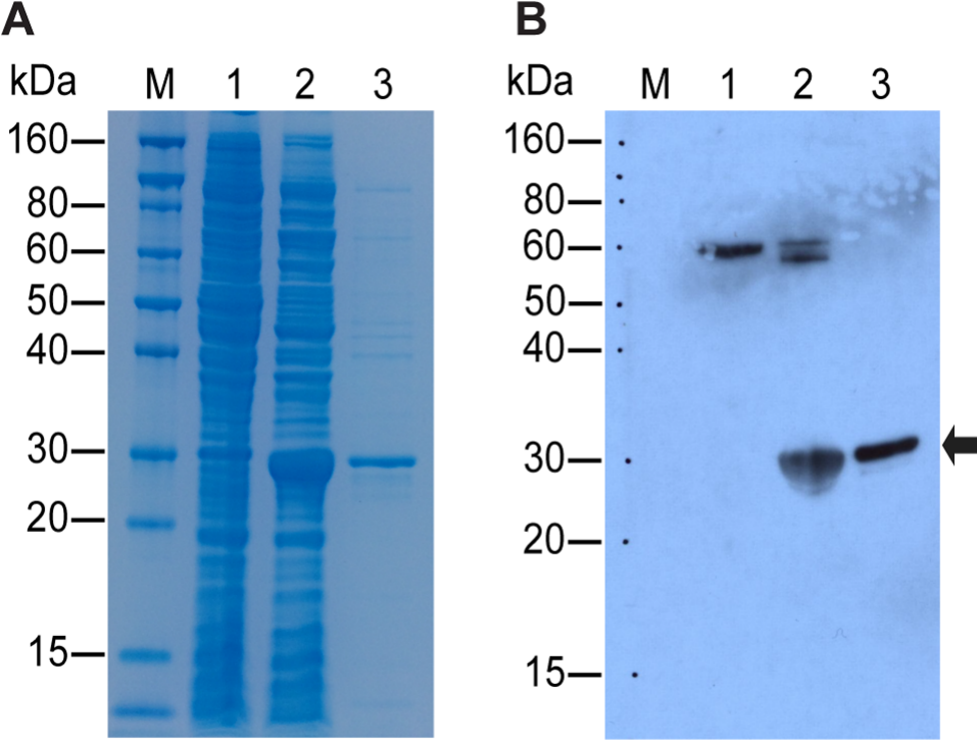
Purification of the domain III subunit of the envelope (E) protein and western blot analysis. (A) Purification of the E protein from the Japanese encephalitis virus (JEV) and (B) western blot analysis using anti-JEV sera. The soluble envelope protein (domain III region) was purified from 50 mL culture using a nickel column. The samples were resolved by 12% gel electrophoresis and transferred onto the PVDF membrane. The blot was probed with JEV-positive sera collected from Japanese encephalitis patients and developed with enhanced chemiluminescent detection reagents. Lanes: M, molecular weight markers; 1, negative control (non-induced cell); 2, whole lysate of the induced cell culture; 3, purified protein fraction from the whole lysate. The arrow on the blot indicates the target protein of size 28.0kDa. The upper bands on lanes 1 and 2 are non-specific and not detected within the purified fraction (lane 3).

Gel electrophoresis and western blotting analyses showed that the over-expressed protein was highly purified in native conditions and reacted strongly with JEV-positive human sera (Fig 3). To obtain sufficient proteins to construct the immunochromatographic test kit, the culture was scaled up to 200 mL and the over-expressed protein was purified using a lab-scale chromatography system (ÄKTA prime plus, GE Healthcare Life Sciences), by which approximately 3.0 mg of highly purified protein was obtained.

### The immunochromatographic test kit

The test kit consisted of a plastic cassette, in which the assembled strip was located, and a 10-mL bottle containing running buffer (Fig 2A). The test was adjusted so that it was completed within 15 min of the running buffer being added to the sample well. In a strong positive sample, two red lines were shown within 5 min at both T and C regions (Fig 2B). In a negative sample, a red line was shown only at C region within 5 min and no color appeared in the T region for at least 20 min. With the IgG IFA as a reference test, the immunochromatographic test had a sensitivity of 84.8% (95/112; 95% confidence interval: 78.3–89.6%) and a specificity of 97.7% (131/134; 95% confidence interval: 93.3–99.5%).

### Field application of the test kit

In the proficiency test, in which all participants tested five serum samples (three JEV-positive, two JEV-negative), all eight provincial IHEs correctly identified the samples and, thus, acquired an overall proficiency score of 100%.

**Fig 4:**
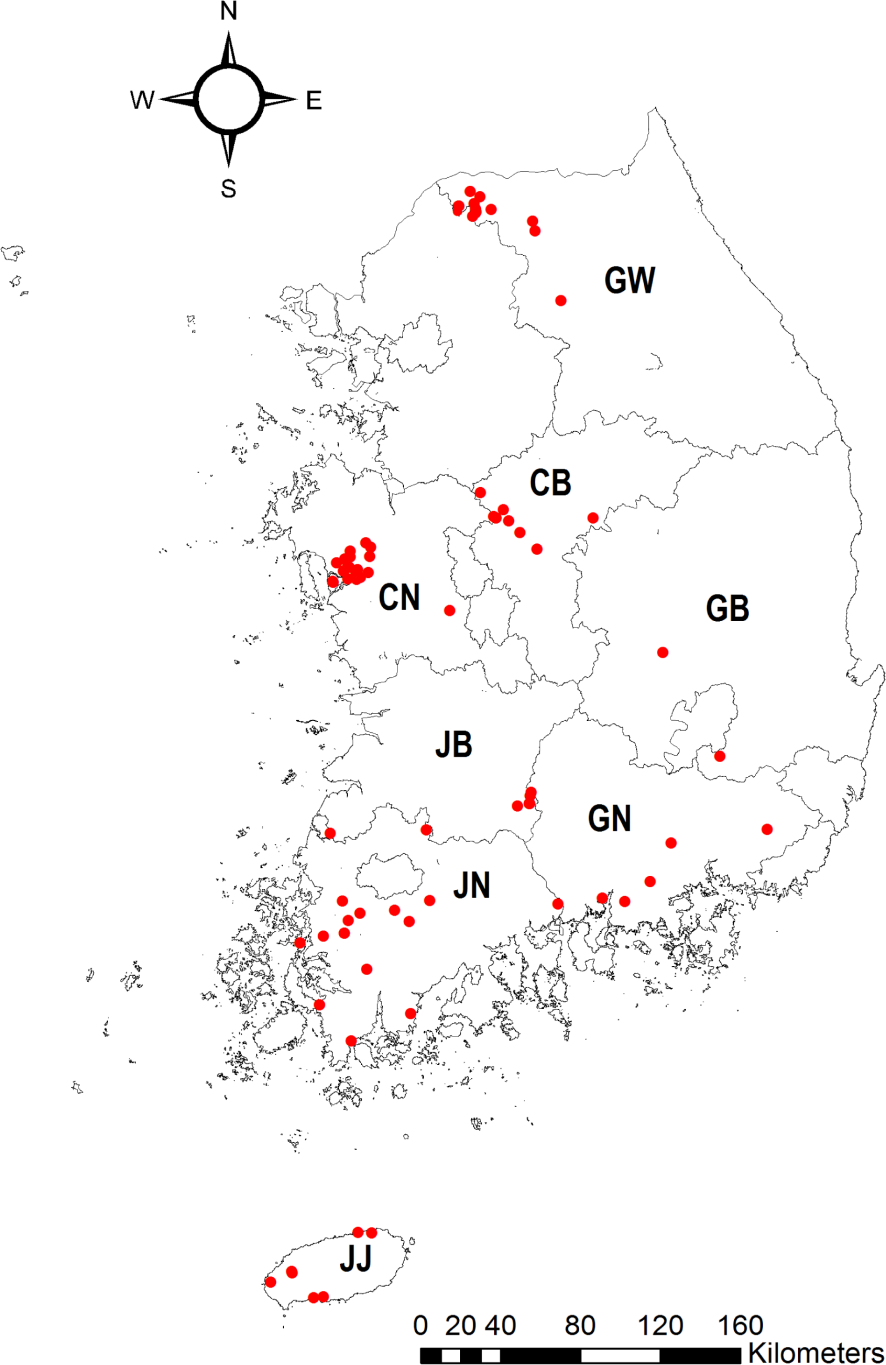
The activity map of Japanese encephalitis virus in Korea in 2012. The locations of pig farms where at least one pig tested positive are marked as closed circles according to their latitude and longitude. CB, Chungbuk province; CN, Chungnam province; JB, Jeonbuk province; JN, Jeonnam province; GN, Gyeongnam province; GB, Gyeongbuk province; GW, Gangwon province; JJ, Jeju province.

During June to October 2012, a total of 1,926 pig sera were collected from 181 pig farms in eight provinces. These pigs had not been vaccinated against JEV and were slaughtered at 6–10 months of age. Every week, each IHE applied the test kit to 10–20 pigs and returned the results to the KNIH. Of the 1,926 samples tested, 228 were JEV-positive (11.8%; 95% confidence interval: 10.5–13.4%) (Table 2). This indicates that at least one pig showed seroconversion to JEV in 77 of the 181 pig farms (42.5%; 95% confidence interval: 35.6–49.8%). The locations of JEV-positive pig farms were marked on a map according to their latitude and longitude (Fig 4).

**Table 2:** Seroconversion to Japanese encephalitis virus in domestic pigs in Korea.

Province	No. of pigs			No. of farms		
	Tested	Positive	Positive rate (%)	Tested	Positive	Positive rate (%)
Chungbuk (CB)	271	38	14.0	23	9	39.1
Chungnam (CN)	360	77	21.4	36	20	55.6
Jeonbuk (JB)	185	9	4.9	25	5	20.0
Jeonnam (JN)	340	45	13.2	27	15	55.6
Gyeongnam (GN)	170	13	7.6	16	6	37.5
Gyeongbuk (GB)	170	5	2.9	17	2	11.8
Gangwon (GW)	270	24	8.9	21	13	61.9
Jeju (JJ)	160	17	10.6	16	7	43.8
Total	1,926	228	11.8	181	77	42.5

The survey was conducted from June to October 2012.

## Discussion

In this study, we developed a new immunochromatographic test for detecting IgG antibodies to JEV in pig serum. Pigs are the major amplifying host of JEV among mammalian species, and seroconversion in pigs is a reliable indicator of virus activity. Although other testing methods such as IFA and ELISA are available, until now the HI test has largely been used for serological testing of JEV infection in pigs, probably because it is cheaper than other methods and requires no specific instruments. One negative aspect of HI is that a number of mice need to be sacrificed to run the test consistently; for example, at our laboratory in the KNIH more than 1,500 suckling mice have been killed to obtain antigens for the HI test every year until 2008. As well as animal welfare issues, the HI test is time-consuming and it is difficult to control the quality of the results between laboratories. ELISA [14, 15] and the latex agglutination test [25] are alternatives to HI, but despite having been developed and evaluated, they are not currently used. In our laboratory, the HI test was replaced with IFA in 2009 and all reagents including antigen slides and secondary antibodies were allocated to IHEs for the proficiency test and routine pig surveillance. While IFA can be completed within 2 h, it requires an expensive fluorescence microscope and results tend to vary between operators.

The colloidal gold particle-based immunoassay has been used since the 1980s and is now applied to study human and animal disease [20–22]. In this study, we constructed three expression vectors of the E protein for the development of a new immunochromatographic test. The E protein is a major candidate for a vaccine target and diagnostic marker in the majority of flaviviruses [1]. However, in our experience, it is difficult to obtain a full-length E protein (500 amino acids) in soluble form in a bacterial expression system. According to previous studies [26], even partial subunits were expressed in insoluble fractions. Thus, we divided the E protein gene into three fragments to be over expressed in soluble form in a bacterial system. Among these, only domain III was expressed in soluble form in an *Escherichia coli* expression system. Other studies have also reported the soluble expression of the domain III region in a bacterial system [27, 28]. In the current study, the domain III region of the E protein was proven to react with anti-JEV antibodies. As a future study, cross-reactivity tests of the recombinant antigen with other flaviviruses would reveal how specific the antigen is to JEV.

Our immunochromatographic test had good sensitivity (84.8%) and specificity (97.7%) compared with an IFA reference test. However, these values should be interpreted with caution because we used IFA as the reference test rather than the gold standard method (plaque reduction neutralization test) and we did not fully test the detection limits of the new test. Nevertheless, the newly developed test could adequately replace traditional tests such as IFA and HI and has several advantages. First, its running time was < 15 min, and it was user-friendly with easily interpreted results, which was proven in the proficiency tests performed prior to field application. For example, five new technicians in five IHEs entered the JE surveillance program in 2012, and all scored 100% in the proficiency test. Second, the new test is cost-effective because it does not need any expensive instruments or trained technicians; indeed, the running cost per test is just 3,500 won (3.1 US dollars as of March 2015).

During the 2012 JE surveillance, 11.8% of the subject pigs were JEV-positive, and these were found on 77 of the 181 pig farms (42.5%). Vaccinated pigs were not included in this study and most pigs intended for meat production in Korea are slaughtered at 6–10 months of age when they reach an appropriate body weight (~100 kg). Moreover, because maternal antibodies persist for 1–3 months [29] and disappear in the majority of pigs by 4–6 months of age [30], the positive results we observed in this study are indicative of infection by JEV. Based on our results, we produced a map that presents the specific locations of JEV activity in 2012. Health authorities could use this resource to accurately identify the places where the virus was active and decide on appropriate control measures such as vector control and public warnings.

We note that the positive JEV rates in each province should not be used for comparing the extent of JEV activity among provinces because sampling was arbitrarily conducted at the point of slaughter. Moreover, the number of pigs and pig farms included in our study was very low in comparison to the total scale observed in Korea in 2012. In further research, a larger sample size and monitoring of the same pig farms will provide information on the temporal and spatial dynamics of JEV in Korea. We also note that an immunochromatographic test for detecting IgM antibodies to JEV could not be produced because monoclonal antibodies to pig IgM were not available at the time the test was developed. Because 2–3 brands of antibodies against pig IgM are now available, a new test for detecting IgM antibodies to JEV could be developed and virus activity could subsequently be detected earlier.

## Conclusion

Here, we presented a novel immunochromatographic test that was developed for the detection of antibodies against JEV in pig serum. The test performed well, with good sensitivity and specificity, and we were able to identify the exact locations where JEV circulates throughout Korea. Therefore, the test kit provides a convenient and effective method for monitoring JEV activity in unvaccinated pigs. In future, the results of the test could help decide on appropriate JEV control measures in a timely manner, and thereby prevent potential outbreaks of JE.

## Supporting Information

S1 File The scale of pigs and pig farms in Korea in 2012. (PDF)

## References

[1] Lindenbach BD, Thiel HJ, Rice CM. Flaviviridae: The viruses and their replication In: Knipe DM, Howley PM, *editors* Fields Virology. 5th edition Philadelphia: Lippincott-Raven Publishers; 2007 pp. 1101–1152.

[2] Endy TP, Nisalak A. Japanese encephalitis virus: ecology and epidemiology. Curr Top Microbiol Immunol. 2002; 267: 11–48. . 12082986 10.1007/978-3-642-59403-8_2

[3] Campbell GL, Hills SL, Fischer M, Jacobson JA, Hoke CH, Hombach JM, et al Estimated global incidence of Japanese encephalitis: a systematic review. Bull World Health Organ. 2011; 89(10): 766–774, 74A–74E. 10.2471/BLT.10.085233 22084515 PMC3209971

[4] Ishikawa T, Yamanaka A, Konishi E. A review of successful flavivirus vaccines and the problems with those flaviviruses for which vaccines are not yet available. Vaccine. 2014; 32(12): 1326–1337. 10.1016/j.vaccine.2014.01.040 24486372

[5] Saito M, Ito T, Amano Y, Takara J, Nakata K, Tamanaha S, et al Trials for risk assessment of Japanese encephalitis based on the serological survey of wild animals In: Ruzek D, editor. Flavivirus encephalitis. Croatia: InTech; 2011 pp. 427–438.

[6] Pyke AT, Williams DT, Nisbet DJ, van den Hurk AF, Taylor CT, Johansen CA, et al The appearance of a second genotype of Japanese encephalitis virus in the Australasian region. Am J Trop Med Hyg. 2001; 65(6): 747–753. . 11791969 10.4269/ajtmh.2001.65.747

[7] Rajendran R, Thenmozhi V, Tewari SC, Balasubramanian A, Ayanar K, Manavalan R, et al Longitudinal studies in South Indian villages on Japanese encephalitis virus infection in mosquitoes and seroconversion in goats. Trop Med Int Health. 2003; 8(2): 174–181. . 12581445 10.1046/j.1365-3156.2003.01003.x

[8] Nidaira M, Taira K, Onodera I, Morikawa T, Itokazu K, Kudaka J, et al Detection of Japanese encephalitis virus antibody in a pig on Yonaguni Island, where all pigs were slaughtered in 1997. Jpn J Infect Dis. 2007; 60(1): 70–71. . 17314436

[9] Arai S, Matsunaga Y, Takasaki T, Tanaka-Taya K, Taniguchi K, Okabe N, et al Japanese encephalitis: surveillance and elimination effort in Japan from 1982 to 2004. Jpn J Infect Dis. 2008; 61(5): 333–338. . 18806337

[10] Yamanaka A, Mulyatno KC, Susilowati H, Hendrianto E, Utsumi T, Amin M, et al Prevalence of antibodies to Japanese encephalitis virus among pigs in Bali and East Java, Indonesia, 2008. Jpn J Infect Dis. 2010; 63(1): 58–60. . 20093765

[11] Borah J, Dutta P, Khan SA, Mahanta J. Epidemiological concordance of Japanese encephalitis virus infection among mosquito vectors, amplifying hosts and humans in India. Epidemiol Infect. 2013; 141(1): 74–80. 10.1017/S0950268812000258 22361257 PMC9152037

[12] Duong V, Sorn S, Holl D, Rani M, Deubel V, Buchy P. Evidence of Japanese encephalitis virus infections in swine populations in 8 provinces of Cambodia: implications for national Japanese encephalitis vaccination policy. Acta Trop. 2011; 120(1–2): 146–150. . 21803019 10.1016/j.actatropica.2011.07.008

[13] Sohn YM. Japanese encephalitis immunization in South Korea: past, present, and future. Emerg Infect Dis. 2000; 6(1): 17–24. . 10653564 10.3201/eid0601.000103 PMC2627978

[14] Xinglin J, Huanchun C, Xiang W, Changming Q. Quantitative and qualitative study of enzyme-linked immunosorbent assay to detect IgG against Japanese encephalitis virus in swine sera. Vet Res Commun. 2005; 29(2): 159–169. . 15730140 10.1023/b:verc.0000047485.85866.6e

[15] Yang DK, Kim BH, Lim SI, Kwon JH, Lee KW, Choi CU, et al Development and evaluation of indirect ELISA for the detection of antibodies against Japanese encephalitis virus in swine. J Vet Sci. 2006; 7(3): 271–275. . 16871022 10.4142/jvs.2006.7.3.271 PMC3242127

[16] Clarke DH, Casals J. Techniques for hemagglutination and hemagglutination-inhibition with arthropod-borne viruses. Am J Trop Med Hyg. 1958; 7(5): 561–573. . 13571577 10.4269/ajtmh.1958.7.561

[17] Koraka P, Zeller H, Niedrig M, Osterhaus AD, Groen J. Reactivity of serum samples from patients with a flavivirus infection measured by immunofluorescence assay and ELISA. Microbes Infect. 2002; 4(12): 1209–1215. . 12467761 10.1016/s1286-4579(02)01647-7

[18] Litzba N, Klade CS, Lederer S, Niedrig M. Evaluation of serological diagnostic test systems assessing the immune response to Japanese encephalitis vaccination. PLoS Negl Trop Dis. 2010; 4(11): e883 10.1371/journal.pntd.0000883 21103412 PMC2982812

[19] Niedrig M, Kursteiner O, Herzog C, Sonnenberg K. Evaluation of an indirect immunofluorescence assay for detection of immunoglobulin M (IgM) and IgG antibodies against yellow fever virus. Clin Vaccine Immunol. 2008; 15(2): 177–181. . 18045884 10.1128/CVI.00078-07 PMC2238043

[20] Peng F, Wang Z, Zhang S, Wu R, Hu S, Li Z, et al Development of an immunochromatographic strip for rapid detection of H9 subtype avian influenza viruses. Clin Vaccine Immunol. 2008; 15(3): 569–574. 10.1128/CVI.00273-07 18199737 PMC2268275

[21] Li XS, Fu F, Lang YK, Li HZ, Wang W, Chen X, et al Development and preliminary application of an immunochromatographic strip for rapid detection of infection with porcine reproductive and respiratory syndrome virus in swine. J Virol Methods. 2011; 176(1–2): 46–52. 10.1016/j.jviromet.2011.06.015 21663767

[22] Jin Q, Yang J, Lu Q, Guo J, Deng R, Wang Y, et al Development of an immunochromatographic strip for the detection of antibodies against Porcine circovirus-2. J Vet Diagn Invest. 2012; 24(6): 1151–1157. 10.1177/1040638712462374 23051825

[23] Cha GW, Cho JE, Ju YR, Hong YJ, Han MG, Lee WJ, et al Comparison of four serological tests for detecting antibodies to Japanese encephalitis virus after vaccination in children. Osong Public Health Res Perspect. 2014; 5(5): 286–291. 10.1016/j.phrp.2014.08.003 25389515 PMC4225649

[24] ExPASy Bioinformatics Resource Portal. Available: http://web.expasy.org/compute_pi/. Accessed 28 November 2014.

[25] Xinglin J, Huanchun C, Qigai H, Xiang W, Bin W, Dexin Q, et al The development and application of the latex agglutination test to detect serum antibodies against Japanese encephalitis virus. Vet Res Commun. 2002; 26(6): 495–503. . 12241102 10.1023/a:1020546626694

[26] Mason PW, Dalrymple JM, Gentry MK, McCown JM, Hoke CH, Burke DS, et al Molecular characterization of a neutralizing domain of the Japanese encephalitis virus structural glycoprotein. J Gen Virol. 1989; 70 (Pt 8): 2037–2049. . 2549181 10.1099/0022-1317-70-8-2037

[27] Chia SC, Leung PS, Liao CP, Huang JH, Lee ST. Fragment of Japanese encephalitis virus envelope protein produced in Escherichia coli protects mice from virus challenge. Microb Pathog. 2001; 31(1): 9–19. . 11427032 10.1006/mpat.2001.0442

[28] Wu SC, Yu CH, Lin CW, Chu IM. The domain III fragment of Japanese encephalitis virus envelope protein: mouse immunogenicity and liposome adjuvanticity. Vaccine. 2003; 21(19–20): 2516–2522. . 12744886 10.1016/s0264-410x(03)00042-2

[29] Geevarghese G, Shaikh BH, Jacob PG, Bhat HR, Pavri KM. Domestic pigs as sentinels to monitor the activity of Japanese encephalitis & West Nile viruses in Kolar district, Karnataka. Indian J Med Res. 1987; 86: 413–418. . 2832323

[30] Scherer WF, Moyer JT, Izumi T. Immunologic studies of Japanese encephalitis virus in Japan. V. Maternal antibodies, antibody responses and viremia following infection of swine. J Immunol. 1959; 83: 620–626. . 14442657

