# Supplementary material for: A Novel Immunochromatographic Test Applied to a Serological Survey of Japanese Encephalitis Virus on Pig Farms in Korea
Source: PLoS One. 2015 May 20;10(5):e0127313. doi: 10.1371/journal.pone.0127313 (PMC4439121; doi:10.1371/journal.pone.0127313)
Supplement: S1 File — (PDF) [file pone.0127313.s001.pdf]

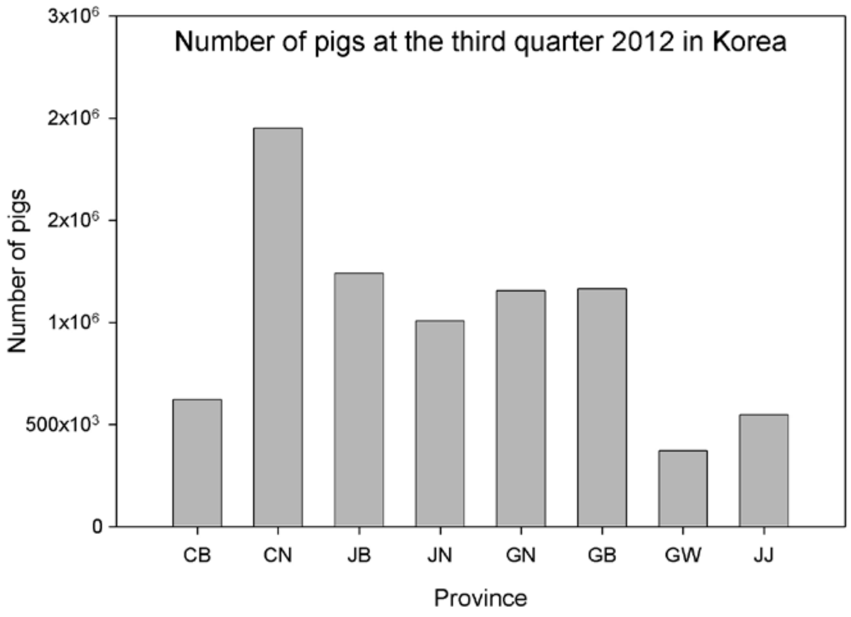

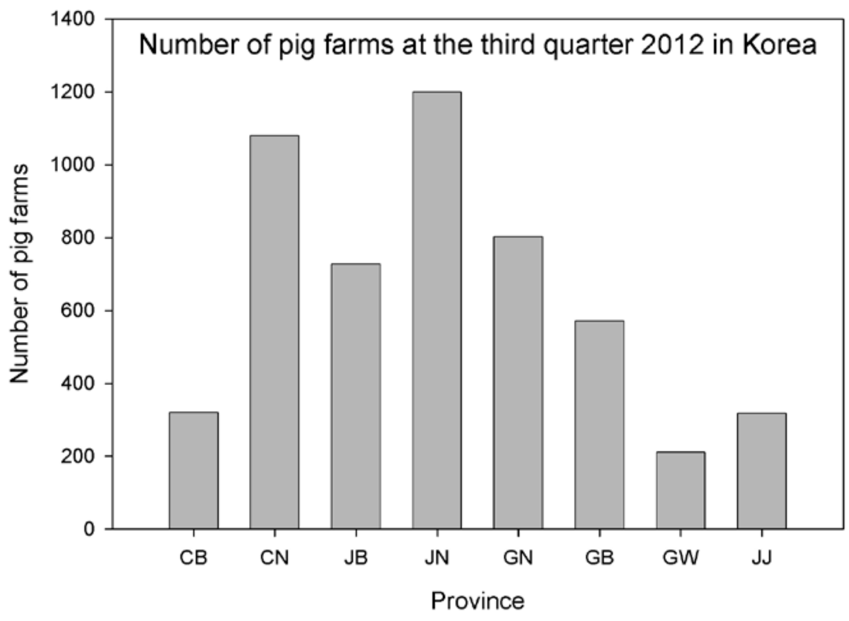

The information was retrieved from the Korean Statistical Information Service (http://kosis.kr) and presented as a bar chart. CB, Chungbuk province; CN, Chungnam province; JB, Jeonbuk province; JN, Jeonnam province; GN, Gyeongnam province; GB, Gyeongbuk province; GW, Gangwon province; JJ, Jeju province.
